# When Delirium Unmasks a Silent Myocardial Infarction

**DOI:** 10.1111/jgs.70281

**Published:** 2025-12-31

**Authors:** Eugenia Casali, Wenxiang Guo, Eleonora Cucini, Francesca Pisani, Alberto Finazzi, Maria Cristina Ferrara, Maurizio Corsi, Elena Pinardi, Chukwuma Okoye, Paolo Mazzola, Giuseppe Bellelli

**Affiliations:** ^1^ Università degli Studi di Milano‐Bicocca School of Medicine and Surgery Monza Italy; ^2^ Centro Studi Dipartimentale sulla Medicina della Complessità e Cure Palliative “Virgilio Floriani” Milano Italy; ^3^ Fondazione IRCCS San Gerardo dei Tintori Hospital, Acute Geriatrics Unit Monza Italy

**Keywords:** acute myocardial infarction, delirium, hip fracture, orthogeriatric

## Abstract

Abbreviations: hs‐TnT: high sensitivity Troponin T; STEMI: ST‐elevation myocardial infarction.

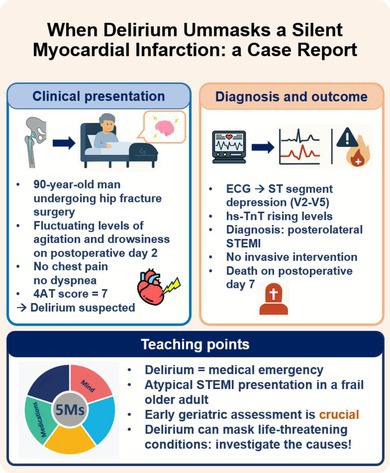

## 1 Patient Story

### 1.1 Setting

Since 2007, a daily orthogeriatric service integrated within the Orthopedics ward supports hip fracture (HF) care in our tertiary hospital [1]. Orthopedic surgeons provide 24/7 surgical and emergency coverage, while a Geriatrician (available on weekdays from 8 a.m. to 2 p.m.) performs comprehensive geriatric assessment (CGA), coordinates treatment, and oversees prevention and discharge planning.

### 1.2 Case Report

A 90‐year‐old man (Mr. P) was admitted to our hospital's Emergency Department (ED) on a Thursday night after sustaining a post‐fall HF. Before the fracture, he lived at home with his wife. He was dependent on assistance for most daily activities, except eating, and ambulated with limited mobility using a walker. According to his wife, he had experienced a progressive cognitive decline over the previous year, which had not been formally evaluated. His past medical history was notable for hypertension, diabetes, chronic kidney and liver disease, and a prior hemicolectomy for colon adenocarcinoma. His at‐home medications included Zofenopril 30 mg, Insulin treatment, Ticlopidine 250 mg, Ezetimibe/Simvastatin 10/20 mg, Trazodone 100 mg at 8 a.m. and 75 mg at 4 p.m.

On admission to ED, all vital signs were within the normal range, indicating clinical stability. Pelvic X‐ray showed a left intertrochanteric HF, while laboratory tests revealed normal hemoglobin levels, elevated serum creatinine (1.9 mg/dL), and mild hyperkalemia (K^+^ = 5.5 mmol/L). To date, his baseline creatinine concentration was 1.3 mg/dL at a previous laboratory workup performed several months before. Electrocardiogram (ECG) revealed a regular sinus rhythm. Mr. P was then admitted to the Orthopedic Unit and underwent osteosynthesis with an intramedullary gamma nail under general anesthesia. Intraoperative hypotension was promptly managed with intravenous saline solution.

On postoperative day 2, serum creatinine levels rose to 2.5 mg/dL, and urea to 139 mg/dL. Intravenous fluids were accordingly increased. Later that day, Mr. P began to alternate between periods of drowsiness and agitation. During a peak episode of agitation, the on‐call physician administered intramuscular Promazine (50 mg IM) without a comprehensive clinical assessment of potential underlying causes. Promazine is a first‐generation phenothiazine antipsychotic, occasionally used to manage agitation in delirium despite limited supporting evidence for its efficacy and safety in this context [2].

**FIGURE 1:**
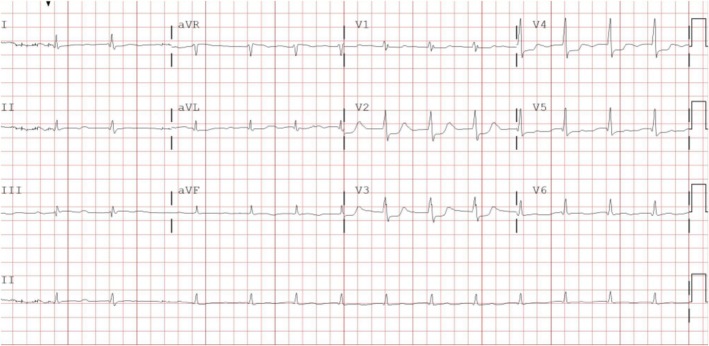
The ECG performed, following the medical examination by the Geriatrician. The ECG reveals sinus rhythm, first‐degree AV block, low voltages in peripheral leads, and ST segment depression in leads V2–V5.

On postoperative Day 3 (Monday), the Geriatrician assumed responsibility for Mr. P and conducted a CGA, as part of standard care. The assessment revealed severe frailty and risk of malnutrition. During the examination, Mr. P presented with moderate psychomotor agitation, characterized by frequent purposeless movements. The 4AT, a rapid bedside screening tool for delirium and cognitive impairment, yielded a score of 7 (range 0–12, with ≥ 4 suggesting possible delirium) [3]. Further evaluation confirmed acute inattention, disorientation, fluctuating alertness, and hyperactive psychomotor behavior, consistent with a diagnosis of delirium according to the *Diagnostic and Statistical Manual of Mental Disorders, Fifth Edition (DSM‐5) criteria* [4]. A comprehensive diagnostic workup was promptly initiated to identify potential precipitating factors, including drugs, electrolyte imbalance, pain, infections, sensory deprivation, intracranial events and urinary or fecal retention. Laboratory and clinical findings allowed all these common causes to be reasonably excluded. However, given Mr. P high cardiovascular risk profile (HEART score for Major Adverse Cardiac Event [5] = 7/10), a cardiac etiology was subsequently considered, despite the previously normal ECG and the absence of typical symptoms such as chest pain or dyspnea [6]. A repeat ECG revealed sinus rhythm with ST‐segment changes (Figure 1), and serial troponin serum measurements demonstrated markedly elevated values, peaking at 12,610 ng/L.

The consultant cardiologist confirmed a diagnosis of acute myocardial infarction, specifically a posterolateral ST‐elevation myocardial infarction (STEMI). Due to the delayed presentation, a conservative (non‐invasive) strategy was recommended. Treatment included intravenous antiplatelet therapy, a P2Y12 inhibitor, and a transdermal nitrate administration. Notably, Mr. P never manifested the typical symptoms of acute myocardial infarction. Despite appropriate medical management, his clinical condition progressively deteriorated over the following days, and he passed away on postoperative Day 7.

## 2 Main Teaching Point

This case emphasizes two key domains of the 5Ms framework: Mind and Medications. Delirium (“Mind”) in older postoperative patients requires urgent recognition and systematic assessment. Mr. P's case illustrates how acute attentive and arousal's abnormalities (drowsiness and/or agitation) may conceal serious underlying conditions, such as acute myocardial infarction, particularly in individuals with prior cognitive decline and/or frailty. Structured bedside evaluation (4AT [3], DSM‐5 [4] criteria) is essential to confirm delirium and prompt appropriate diagnostic workup.

Regarding “Medications,” a comprehensive clinical evaluation is critical before initiating pharmacologic treatment for agitation in older adults. Symptomatic therapy without identifying underlying triggers may delay life‐threatening diagnoses. In this case, intramuscular Promazine—a first‐generation antipsychotic with limited evidence for delirium management [2]—was administered emergently by the on‐call orthopedic surgeon. Geriatric Medicine consultation could have supported safer, evidence‐based alternatives and optimized overall care.

## 3 Discussion

This case illustrates the interplay between cognitive and pharmacologic domains in geriatric care and highlights the importance of interprofessional collaboration among specialists in managing complex postoperative patients. Shared decision‐making and awareness of the 5Ms framework are crucial to improving diagnostic precision, minimizing harm, and ensuring patient‐centered outcomes.

In older adults undergoing HF surgery, acute cognitive changes, drowsiness, or agitation should not be regarded as merely postoperative issues or inevitable sequelae of surgery, but as medical emergencies that may signal life‐threatening conditions. From this perspective, delirium should be systematically screened for in all patients with HF, to prevent underrecognition [7] and delayed diagnosis. Additionally, clinical management should extend beyond the control of agitation to include the investigation of potential underlying causes. Focusing solely on symptomatic relief risks masking serious precipitating conditions and delaying definitive treatment [8].

In this case report, delirium was suspected early by the orthopedic team, yet management focused primarily on calming agitation, while comprehensive assessment was deferred. The subsequent geriatric evaluation reframed the presentation as a delirium syndrome and prompted further diagnostic workup, ultimately revealing an acute myocardial infarction in the absence of classical symptoms. Evidence suggests that delirium can represent the sole manifestation of acute myocardial ischemia [5], and such atypical presentations are associated with increased mortality compared with cases without delirium [9].

Healthcare providers should therefore be trained to recognize delirium promptly, investigate its potential causes systematically, and engage the expertise of a Geriatrician early to improve diagnostic accuracy and clinical outcomes [10].

## Author Contributions

**E.Ca.:** acquisition of subject's information and data, review of the literature, preparation of manuscript, preparation of figures. **W.G.:** acquisition of subject's information and data, review of the literature, preparation of manuscript, preparation of figures. **E.Cu.:** review of the literature, interpretation of data, preparation of manuscript. **A.F.:** interpretation of data, preparation of manuscript. **M.C.F.:** interpretation of data, preparation of manuscript. **M.C.:** interpretation of data, preparation of manuscript. **E.P.:** interpretation of data, manuscript revision according to the comments. **C.O.:** interpretation of data, preparation of manuscript. **P.M.:** study concept and design, interpretation of data, preparation of manuscript, manuscript revision. **G.B.:** study concept and design, interpretation of data. All authors have approved the final version of the manuscript.

## Funding

The authors have nothing to report.

## Conflicts of Interest

The authors declare no conflicts of interest.
